# Morphology and molecular phylogeny of a marine interstitial tetraflagellate with putative endosymbionts: *Auranticordis quadriverberis *n. gen. et sp. (Cercozoa)

**DOI:** 10.1186/1471-2180-8-123

**Published:** 2008-07-22

**Authors:** Chitchai Chantangsi, Heather J Esson, Brian S Leander

**Affiliations:** 1 Canadian Institute for Advanced Research, Program in Integrated Microbial Biodiversity, Department of Zoology, University of British Columbia, Vancouver, BC, V6T 1Z4, Canada; 2 Canadian Institute for Advanced Research, Program in Integrated Microbial Biodiversity, Department of Botany, University of British Columbia, Vancouver, BC, V6T 1Z4, Canada

## Abstract

**Background:**

Comparative morphological studies and environmental sequencing surveys indicate that marine benthic environments contain a diverse assortment of microorganisms that are just beginning to be explored and characterized. The most conspicuous predatory flagellates in these habitats range from about 20–150 μm in size and fall into three major groups of eukaryotes that are very distantly related to one another: dinoflagellates, euglenids and cercozoans. The Cercozoa is a diverse group of amoeboflagellates that cluster together in molecular phylogenies inferred mainly from ribosomal gene sequences. These molecular phylogenetic studies have demonstrated that several enigmatic taxa, previously treated as Eukaryota *insertae sedis*, fall within the Cercozoa, and suggest that the actual diversity of this group is largely unknown. Improved knowledge of cercozoan diversity is expected to help resolve major branches in the tree of eukaryotes and demonstrate important cellular innovations for understanding eukaryote evolution.

**Results:**

A rare tetraflagellate, *Auranticordis quadriverberis *n. gen. et sp., was isolated from marine sand samples. Uncultured cells were in low abundance and were individually prepared for electron microscopy and DNA sequencing. These flagellates possessed several novel features, such as (1) gliding motility associated with four bundled recurrent flagella, (2) heart-shaped cells about 35–75 μm in diam., and (3) bright orange coloration caused by linear arrays of muciferous bodies. Each cell also possessed about 2–30 pale orange bodies (usually 4–5 μm in diam.) that were enveloped by two membranes and sac-like vesicles. The innermost membrane invaginated to form unstacked thylakoids that extended towards a central pyrenoid containing tailed viral particles. Although to our knowledge, these bodies have never been described in any other eukaryote, the ultrastructure was most consistent with photosynthetic endosymbionts of cyanobacterial origin. This combination of morphological features did not allow us to assign *A. quadriverberis *to any known eukaryotic supergroup. Thus, we sequenced the small subunit rDNA sequence from two different isolates and demonstrated that this lineage evolved from within the Cercozoa.

**Conclusion:**

Our discovery and characterization of *A. quadriverberis *underscores how poorly we understand the diversity of cercozoans and, potentially, represents one of the few independent cases of primary endosymbiosis within the Cercozoa and beyond.

## Background

Marine benthic environments contain a diverse assortment of microorganisms that are still just beginning to be explored and characterized [1,2]. The challenges associated with extracting and enumerating benthic microorganisms and the extreme variation of physical and chemical factors associated with the benthos have limited our understanding of these ecosystems [2]. Nonetheless, both comparative morphological studies and environmental sequencing surveys have revealed a great deal of microeukaryotic diversity within the interstitial spaces of marine sediments [3-16]. The most conspicuous predatory flagellates in these habitats range from about 20–150 μm in size and fall into three major groups of eukaryotes that are very distantly related to one another: dinoflagellates, euglenids and cercozoans.

The Cercozoa is a large and diverse group of amoeboflagellates, with tubular mitochondrial cristae, that cluster together in molecular phylogenies inferred mainly from ribosomal gene sequences (small and large subunit rDNA) [4,17-20]. Although a robust morphological synapomorphy is currently lacking for the group, members of the Cercozoa do share novel molecular traits (i.e. molecular synapomorphies), such as the insertion of one or two amino acid residues between the monomer tracks of highly conserved polyubiquitin genes [17]. Nonetheless, molecular phylogenetic studies have demonstrated that several enigmatic taxa, previously treated as Eukaryota *insertae sedis*, fall within the Cercozoa, such as *Allantion*, *Allas*, *Bodomorpha *and *Spongomonas *[21]; *Cryothecomonas *[22]; *Ebria *[23]; *Gymnophrys *and *Lecythium *[24]; *Massisteria *[25]; *Metopion *and *Metromonas *[4]; *Proleptomonas *[26]; and *Protaspis *[8]. Moreover, environmental sequencing surveys have demonstrated several cercozoan subclades without clear cellular identities, suggesting that the actual diversity of this group is composed of thousands of uncharacterized lineages [4]. It must also be emphasized that morphological information from cercozoans, especially at the ultrastructural level, is largely absent from the literature. Accordingly, we characterized the ultrastructure and molecular phylogeny of a highly unusual and rarely encountered tetraflagellate, *Auranticordis quadriverberis *n. gen. et sp. (Cercozoa), isolated from sand samples collected in a marine tidal flat. Uncultured cells were individually isolated and prepared for DNA extraction (performed twice on different days, n = 5 and n = 1), transmission electron microscopy (TEM, n = 2) and scanning electron microscopy (SEM, n = 25). This approach enabled us to describe the ultrastructure of intracellular pigmented bodies within *A. quadriverberis *that are most likely photosynthetic endosymbionts derived from cyanobacterial prey.

## Results

### General morphology and behaviour

**Figure 1:**
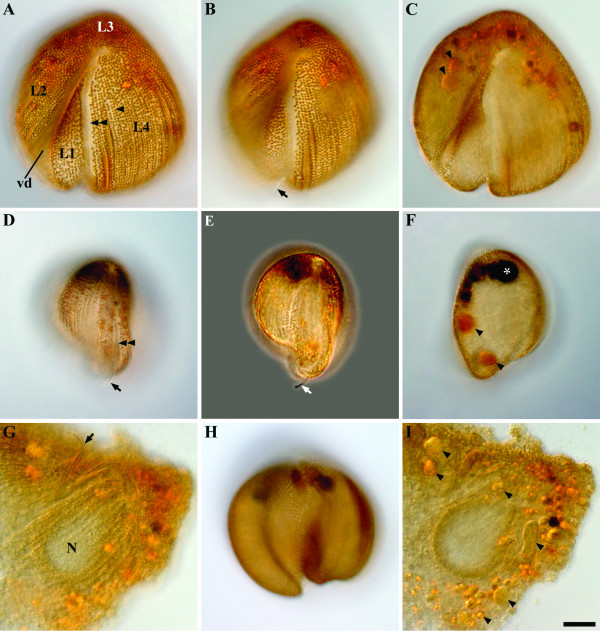
Light micrographs (LM) of *Auranticordisquadriverberis *n. gen. et sp. showing cell color, main cytoplasmic components, and variation in cell shape. **A. **Differential interference contrast (DIC) image focused on rows of longitudinally arranged orange muciferous bodies (arrowhead), the ventral groove (double arrowhead), lobe 1 (L1), a ventral depression (vd), L2, L3, and L4. **B. **An inverted heart-shaped cell with visible flagella (arrow) emerging from the posterior region of the ventral groove. **C. **A flattened cell showing larger pale orange bodies (putative primary endosymbionts, arrowheads) distributed in the anterior end of the cell. **D. **DIC image showing the position of the ventral groove (double arrowhead) with flagella (arrow) relative to a prominent L1 and L4. **E. **Phase contrast micrograph demonstrating the distal end of the flagella emerging from the ventral groove. **F. **DIC micrograph showing black bodies (asterisk) accumulated at the anterior end of the cell and two pale orange bodies (putative primary endosymbionts, arrowheads). **G. **A squashed cell showing the anterior nucleus (N) and flagella (arrow). **H. **DIC micrograph showing a cell with prominent lobes. **I. **A squashed cell showing variation in the shape and size of the pale orange bodies (putative primary endosymbionts, arrowheads). (A-I, Bar = 10 μm).

**Figure 2:**
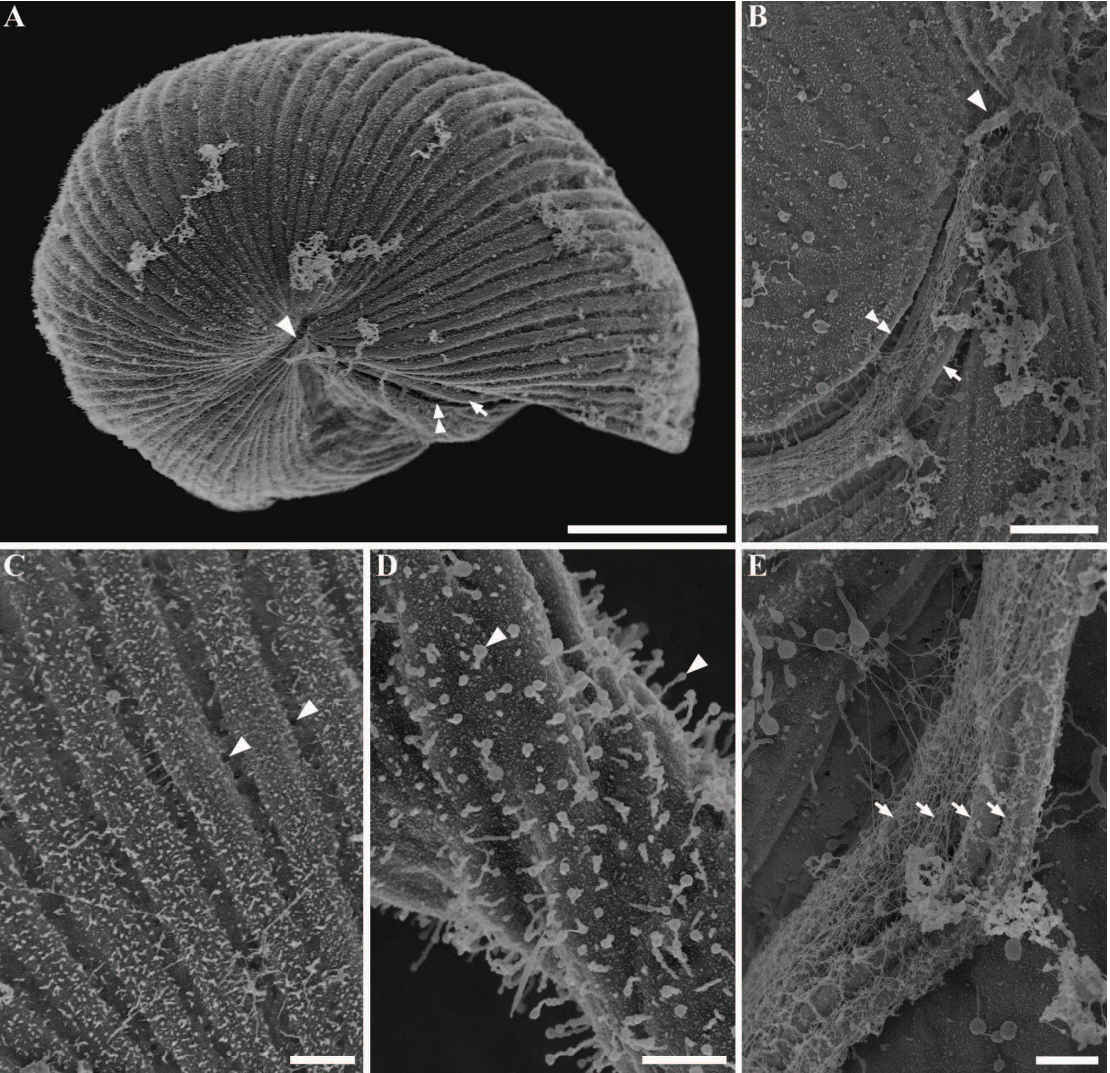
Scanning electron micrographs (SEM) of *Auranticordis quadriverberis *n. gen. et sp. **A. **An anterior view of the cell showing the anterior apex (arrowhead), ventral groove (double arrowhead) and flagella (arrow) (Bar = 10 μm). **B. **A higher magnification view of the anterior end of the cell (arrowhead) showing the flagella (arrow) within the ventral groove (double arrowhead) (Bar = 2 μm). **C. **High magnification view of the ridges showing several tiny pores (arrowheads) in the grooves (Bar = 1 μm). **D. **High magnification view of secreted mucus (arrowheads) (Bar = 0.5 μm). **E. **High magnification view of the ventral groove showing all four flagella (arrows) bundled together and covered in hairs (Bar = 0.5 μm).

**Figure 3:**
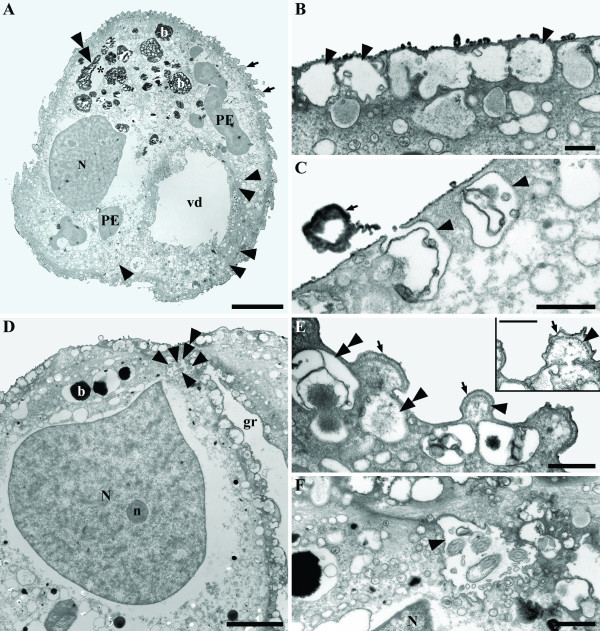
Transmission electron micrographs (TEM) of *Auranticordis quadriverberis *n. gen. et sp. **A. **Low magnification view showing the main cellular components: black bodies (b), nucleus (N), pale orange bodies (putative primary endosymbionts, PE), a degraded PE (double arrowhead) surrounded by sac-like vesicles (asterisk), surface ridges (arrows), and the ventral depression (vd) (Bar = 10 μm). **B. **Section through the surface showing a row of muciferous bodies (arrowheads) containing (orange) amorphous material. Each muciferous body is about 500–900 nm in diameter (Bar = 0.5 μm). **C. **High magnification view of muciferous bodies (arrowheads) and secreted mucus (arrow) (Bar = 0.5 μm). **D. **Section through the anterior region of the cell showing black bodies (b), the flagellar pocket (double arrowhead), four flagella (arrows), a nucleolus (n), a pointed nucleus (N), and the ventral groove (gr) (Bar = 5 μm). **E. **High magnification section through the surface ridges (arrow) showing underlying microtubules (arrowhead) and muciferous bodies (double arrowheads) (Bar = 0.5 μm). An inset showing a magnified view of a surface ridge (arrow) with a row of microtubules underneath (arrowhead) (Bar = 0.5 μm). **F. **Transverse section showing all four flagella within a flagellar pocket (arrowhead) near the nuclear anterior projection (N) (Bar = 1 μm).

*Auranticordis quadriverberis *was able to glide slowly using four tightly bundled flagella that were oriented posteriorly. The cells of *A. quadriverberis *were also able to change shape, albeit only slightly, and could be prominently lobed, heart-shaped or ovoid (Figures 1A–F, H). In general, the cells had a narrower anterior apex and an expanded posterior end and were composed of four major lobes (L): L1, L2, L3, and L4 (Figure 1A). L1 was smaller than other three lobes and was separated from L2, to the right, by a ventral depression (vd) and separated from L4, to the left, by a ventral groove (gr) that contained the four recurrent flagella (Figure 1A). Apart from differences in cell shape and the effects of cell plasticity, there was also variation in the size of different individuals, ranging from 35–75 μm in diam. (n = 65). The cells were conspicuously orange in color, caused mostly by the presence of linear arrays of tiny orange muciferous bodies that were distributed over the entire surface of the cell (Figures 1A–B). Microscopical observations indicated that these bodies secrete sticky mucilage when the cells are disturbed, suggesting that the bodies function for adhesion to the substratum. TEM micrographs showed that the muciferous bodies were small compartments (780 nm in diam.) positioned underneath the cell membrane and filled with amorphous material that was secreted as mucilaginous strands (Figures 2D, 3B–C). The surface of *A. quadriverberis *was also corrugated and consisted of over 80 longitudinal ridges that spanned from the anterior apex to the posterior end (Figures 2A–C). The grooves between the ridges contained numerous tiny pores through which the mucilage from the muciferous bodies was secreted (Figure 2C). TEM sections through the cell surface also demonstrated a single row of microtubules positioned beneath each ridge (Figure 3E). No test or cell wall was present.

**Figure 4:**
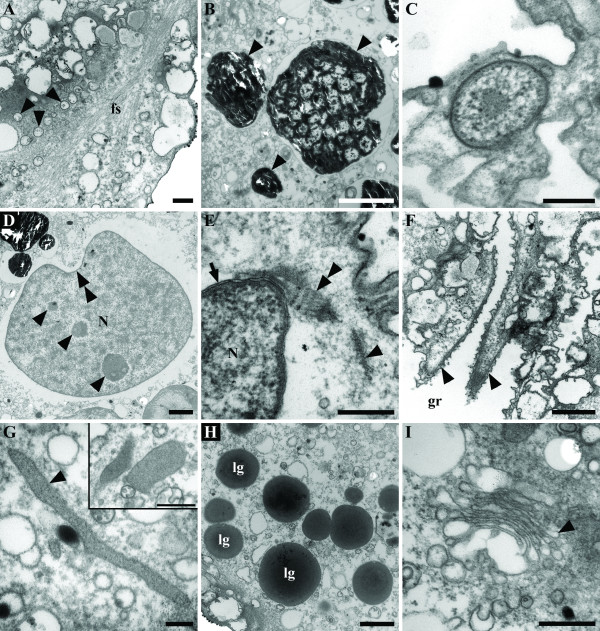
Transmission electron micrographs (TEM) of *Auranticordis quadriverberis *n. gen. et sp., showing different cytoplasmic components. **A. **High magnification TEM showing a vacuolated cytoplasm (arrowheads) and fibrous material (fs) distributed beneath the cell periphery (Bar = 0.5 μm). **B. **High magnification view of the black inclusions (arrowheads) (Bar = 2 μm). **C. **An ingested bacterium found within cytoplasm of *A. quadriverberis *(Bar = 0.25 μm). **D. **A section through the nucleus (N) showing nucleoli (arrowheads) and an invaginated area (double arrowhead) (Bar = 2 μm). **E. **High magnification TEM showing the nuclear envelope (arrow), the nucleus (N), and a striated band (double arrowhead) positioned between the nuclear tip and a microtubular root (arrowhead) (Bar = 0.5 μm). **F. **Tangential section through the flagella (arrowheads) lying within the ventral groove (gr) (Bar = 1 μm). **G. **A putative mitochondrion positioned near the cell periphery (Bar = 0.2 μm). An inset showing two putative mitochondria (Bar = 0.5 μm). **H. **TEM showing lipid globules (lg) near the posterior part of the cell (Bar = 1 μm). **I. **High magnification view of a Golgi apparatus (Bar = 0.5 μm).

The four flagella of *A. quadriverberis *originated from an anterior flagellar pocket and nestled tightly within the ventral groove, making them nearly invisible under the light microscope (Figures 1B, 1D–E, 1G, 2A–B, 2E, 4F). Electron microscopy demonstrated that the flagella were arranged in two pairs and covered with flagellar hairs or mastigomenes (Figure 2E). Except for very slight differences in length, all four flagella were morphologically identical and slightly longer than the cell (Figures 1B, D–E). The flagella were also homodynamic and associated with gliding motility along the substratum. Pseudopodia were not observed.

### Main cytoplasmic components

*Auranticordis quadriverberis *contained a large nucleus (15–20 μm in diam.) situated in the anterior region of the cell (Figures 3A, 3D). Although the position of the nucleus in living specimens cannot be readily seen under the light microscope, the nucleus is visible in compressed cells as a comparatively clear area (Figures 1G, 1I). TEM sections demonstrated the nuclear envelope and a few prominent nucleoli (Figures 3A, 3D, 4D–E). The nucleus was pointed at the anterior end and was connected to a striated band near the basal bodies and microtubular roots (Figures 3D, 4D–E). Moreover, bundles of (non-microtubular) fibrous material were also observed within the cytoplasm near the cell periphery (Figure 4A).

**Figure 5:**
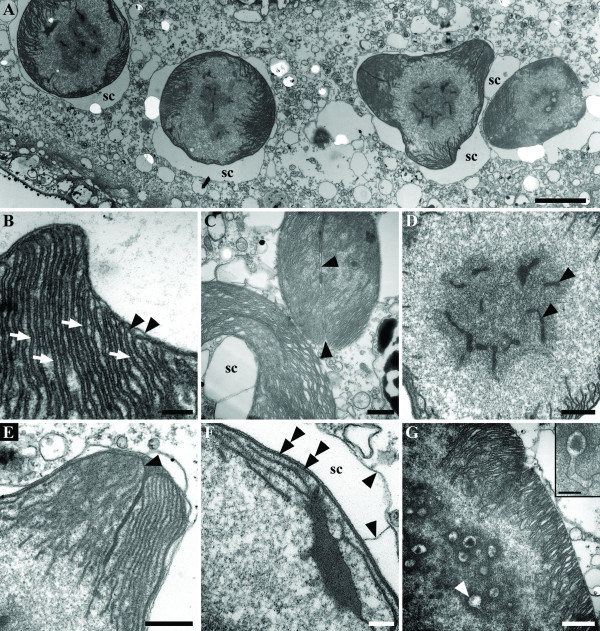
Transmission electron micrographs (TEM) showing the ultrastructure of putative primary endosymbionts in *Auranticordis quadriverberis *n. gen. et sp. **A. **Low magnification TEM showing four putative endosymbionts, each surrounded by sac-like vesicles (sc) defined by an outer membrane (Bar = 2 μm). **B. **High magnification TEM showing two enveloping inner membranes (arrowheads) and thylakoids (arrows) that are continuous with the innermost enveloping membrane (Bar = 0.2 μm). **C. **TEM showing the thylakoids, the sac-like vesicle (sc), and a cleavage furrow indicative of division (arrowheads) (Bar = 0.5 μm). **D. **High magnification TEM showing the central core of an endosymbiont containing viral particles (arrowheads) (Bar = 0.5 μm). **E. **High magnification TEM showing a pronounced invagination of the innermost enveloping membrane (arrowhead) (Bar = 0.5 μm). **F. **High magnification TEM showing the membrane (arrowheads) that defines the sac-like vesicle (sc) and the two innermost enveloping membranes (double arrowheads) (Bar = 0.2 μm). **G. **TEM showing viral particles (arrowhead) consisting of a polygonal head and tail, and positioned within the core of an endosymbiont (Bar = 0.5 μm). An inset showing a complete tailed viral particle (Bar = 0.2 μm).

**Figure 6:**
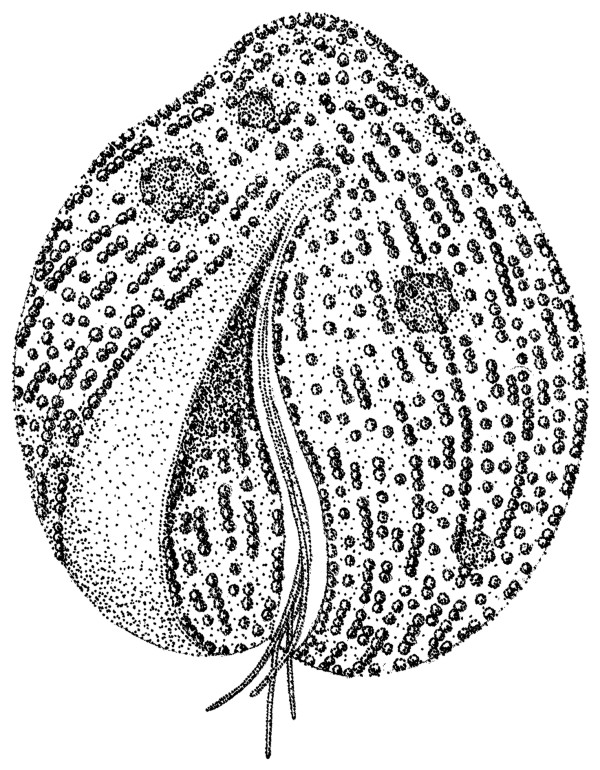
A schematic line drawing of *Auranticordis quadriverberis *n. gen. et sp. The line drawing was constructed from light micrographs and showing a lobed cell, rows of tiny orange muciferous bodies (small circles), four flagella within ventral groove, a ventral depression (lightly stippled area to the left of the flagella), and four putative primary endosymbionts (large shaded circles).

The cells of *A. quadriverberis *also contained an accumulation of black material near the anterior part of the cell, lipid globules and Golgi bodies (Figures 1C, 1F, 1I, 4H–I). Although mitochondria with tubular cristae were not definitively observed, several elongated bodies that were highly reminiscent of acristate mitochondria were found near the periphery of the cell (Figures 3A, 4G). The cells also contained 2–30 pale orange bodies that were variable in shape and usually about 4–5 μm in diam.; however, some of these bodies were 14 μm long (Figures 1C, 1F, 1I, 3A, 5A–G, 6). The pale orange bodies were distributed throughout the cell, but were most abundant in the anterior region of the cell. Each pale orange body was enveloped by two tightly pressed inner membranes and surrounded by sac-like vesicles (Figures 5A, 5C, 5F). The innermost membrane invaginated into the lumen of the body and formed several unstacked thylakoids around the periphery (Figures 5A–C, 5E). The sac-like vesicles occasionally butted together to form perpendicular partitions outside of the two inner membranes (Figure 5F). The central core of the pale orange bodies was devoid of membranes and contained a central electron dense region containing tailed viral particles (Figures 5D, 5G).

### Molecular phylogenetic position of auranticordis

Phylogenetic analyses of a 69-taxon dataset representing all major groups of eukaryotes showed *A. quadriverberis *branching within the Cercozoa with very strong statistical support (data not shown). This cercozoan clade, comprised of *Chlorarachnion reptans*, *Cryothecomonas aestivalis*, *C. longipes*, *Ebria tripartita*, *Euglypha rotunda*, *Heteromita globosa *and *A. quadriverberis*, was strongly supported in both maximum likelihood (ML) and Bayesian analyses (ML boostrap = 100 and Bayesian posterior probabilities = 1.00; data not shown). A more comprehensive analysis of 981 homologous positions in 126 cercozoan SSU rDNA sequences, including several shorter environmental sequences, placed *A. quadriverberis *near *Pseudopirsonia mucosa *(a parasitic nanoflagellate of diatoms) and two unidentified cercozoans with 1.00 Bayesian posterior probabilities (data not shown). Accordingly, we performed phylogenetic analyses of 1,571 positions in 32 cercozoan taxa that excluded the shortest environmental sequences and included the closest relatives of *A. quadriverberis *in the 126-taxon alignment.

**Figure 7:**
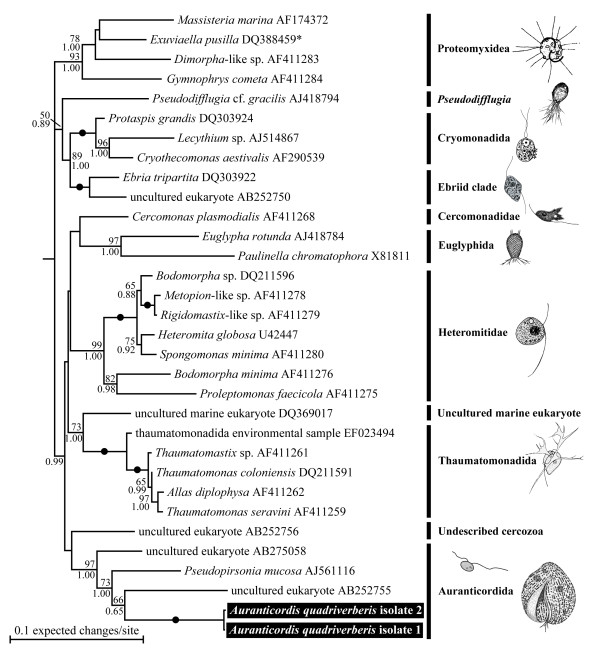
Maximum likelihood (ML) tree (-ln *L *= 10139.70214) inferred from 32 SSU rDNA sequences, 1,571 unambiguously aligned sites and a GTR+I+G+8 model of nucleotide substitutions. Numbers above the branches denote PhyML bootstrap percentages, and numbers below the branches denote Bayesian posterior probabilities. Black circles denote PhyML bootstrap percentages and posterior probabilities of 100% and 1.00, respectively. Line drawings were modified from the following sources: *Massisteria marina *[10], *Pseudodifflugia gracilis *[70], *Cryothecomonas *sp. [71], *Ebria tripartita *[72], *Cercomonas *sp. [73], *Euglypha alveolata *[74], *Heteromita globosa *[34], *Thaumatomonas lauterborni *[75], and *Pseudopirsonia *sp. [76]. The asterisk next to sequence [GenBank:DQ388459] was derived from an environmental sequencing survey and was listed in GenBank as the dinoflagellate *Exuviaella pusilla *by Lin et al. [77].

Figure 7 illustrates the phylogenetic analyses of the 32-taxon dataset. Like in the analyses of 126 taxa, the two different isolates of *A. quadriverberis *clustered with two uncultured eukaryotes and *P. mucosa *(Figure 7). A subclade consisting of *A. quadriverberis*, *P. mucosa *and environmental sequence AB252755 was recovered with a posterior probability of 1.00 and 73% PhyML bootstrap value. A more inclusive clade consisting of *A. quadriverberis*, *P. mucosa *and environmental sequences AB252755 and AB275058 received high statistical support (posterior probability of 1.00 and PhyML bootstrap value of 97%) (Figure 7). Members of this clade also shared a derived molecular character within the context of 160 cercozoan sequences covering representatives from all known cercozoan subclades: namely, the substitution of cytosine (C) for thymine (T) at position 324 (with reference to the complete SSU rDNA sequence of *Cercomonas *sp.; GenBank accession no. AF411266, culture ATCC PRA-21) in Helix 12, based on the predicted secondary structure of the SSU rRNA gene in *Palmaria palmata *[27].

## Discussion

### Comparative morphology

The distinctly orange color of *A. quadriverberis *sets these flagellates apart from other organisms living in the same benthic environment. To our knowledge, similar organisms have not been recorded previously [3,9-12,28]; however, the orange color of *A. quadriverberis *is most reminiscent of the anoxic euglenozoan *Calkinsia aureus *[29].

The presence of four recurrent flagella in *A. quadriverberis *is another distinctive feature. Most cercozoans possess two flagella, although *Cholamonas cyrtodiopsidis *also has four flagella that are inserted subapically [30,31]. The flagella of *C. cyrtodiopsidis *form two symmetrical pairs comprising one long and one stubby flagellum [30,31]. This flagellar organization differs from *A. quadriverberis*, which has two pairs of tightly bundled flagella originating from the same flagellar reservoir. *Cholamonas cyrtodiopsidis *was assigned to the Cercomonadida due to possession of a microbody and kinetid architecture that is similar to some species of *Cercomonas *[30,31]. Although both *A. quadriverberis *and *C. cyrtodiopsidis *possess four flagella, this character state is unlikely to be synapomorphic for these species: *A. quadriverberis *inhabits marine sand, whereas *C. cyrtodiopsidis *inhabits the intestines of diopsid flies [30]. Moreover, the distinctive features present in one species tend not to be shared by the other (e.g. the paranuclear bodies found in *C. cyrtodiopsidis *are not present in *A. quadriverberis*). Because the phylogenetic position of *C. cyrtodiopsidis *has not yet been evaluated with molecular phylogenetic data, our ability to infer the evolution of the tetraflagellated state within the Cercozoa is limited.

The flagella of *A. quadriverberis *are covered by hairs, and although this stands in contrast to the smooth flagella described in most other cercozoans, such as *Cercomonas *and *Proleptomonas *[31], the hairs could be homologous to those described in the predatory soil-dwelling flagellate *Aurigamonas solis *[16,32]. The four flagella of *A. quadriverberis *were also recurrent and homodynamic during gliding motility, which is unlike the heterodynamic flagella of most other interstitial cercozoans (e.g. *Cercomonas*, *Heteromita*, *Katabia*, *Proleptomonas*, and *Protaspis*) [8,31]. The gliding cells of *A. quadriverberis *were plastic and capable of slow changes in shape that was somewhat similar to that found in euglenids [33]. This plasticity is probably generated by the row of microtubules locating underneath the cell membrane (Figure 3E).

The nucleus of *A. quadriverberis *is difficult to see in living cells, which is also unlike most other cercozoans (e.g. *Aurigamonas*, *Cercomonas*, *Ebria*, *Euglypha*, *Heteromita*, *Protaspis*, *Thaumatomastix*, and *Thaumatomonas*) [8,10,16,23,34]. The bloated shape of the cell and the dense distribution of minute orange muciferous bodies that subtend the entire surface of the cell obscured the nucleus. The ultrastructure of the nucleus is similar to that of other cercozoans (e.g. contained several nucleoli) [8,16,35-37]; however, *A. quadriverberis *lacked permanently condensed chromosomes like those found in *Cryothecomonas*, *Ebria*, and *Protaspis *[8,16,23,35,37,38]. The shape of the nucleus in *A. quadriverberis *was indented at one side, a feature also noticed in the nucleus of *Protaspis grandis *[8], and had a prominent anterior projection oriented towards the flagellar pocket. An anterior projection was also observed in the nucleus of *Cercomonas*; in both genera, the anterior projection was associated with a broad striated band and the ventral (posterior) roots of the anterior and posterior flagella (VP) [31,36]. However, the characteristic microtubular cone present in *Cercomonas *[31,36] was not observed in *A. quadriverberis*.

The cytoplasm of *A. quadriverberis *contained lipid globules, Golgi bodies and muciferous bodies. The muciferous bodies were compartments organized in linear arrays and filled with an amorphous matrix that appeared bright orange under the light microscope. Extrusomes like these have also been reported in *C. armigera *as a minute peripheral concavities filled with a homogeneous matrix [37]. Other types of extrusomes that have been found in different cercozoan species, such as trichocysts, microtoxicysts, kinetocysts and osmiophilic bodies, [8,31,36], were absent in *A. quadriverberis*. The lipid globules varied considerably in size and were most abundant in the posterior region of *A. quadriverberis*. These globules were reminiscent of those described in *Protaspis *[8]. Although the mode of feeding in *A. quadriverberis *was not clearly observed, evidence of ingested bacteria was observed within its cytoplasm (Figure 4C).

The cytoplasm of *A. quadriverberis *was highly vacuolated and looked similar to the cytoplasm described in *Cryothecomonas armigera *and *Protaspis grandis *[8,37]. The anterior part of the cell, however, contained black bodies similar to those that have been observed in other distantly related eukaryotes, such as some semi-anoxic euglenids and ciliates. Moreover, distinct mitochondria with tubular cristae, which are characteristic of other cercozoans, were not found in *A. quadriverberis*. Putative mitochondria were, however, observed around the cell periphery (Figure 4G), and the lack of cristae in these organelles reflects either degenerate mitochondria associated with a low-oxygen environment or fixation artifact [39]. The size of the putative mitochondria ranged between 135–185 nm long, which is smaller than the mitochondria described in most cercozoans. For example, the mitochondria of *Aurigamonas solis *are about 630 nm [16], the mitochondria of *Cercomonas *are about 485 nm [36], the mitochondria of *Cryothecomonas longipes *are about 280 nm [40], and the mitochondria of *P. grandis *are about 500 nm [8]. Although the implementation of fluorescent stains, like Mitotracker, could help establish the identity of these structures [41], this approach is limited by the scarcity of these organisms in natural environments and the unpredictability of finding them in our samples.

### Putative primary endosymbionts

Several light orange bodies about 4–14 μm in diam. were distributed within the cell and were especially abundant towards the anterior end of the cell. Although the ultrastructure of these pigmented bodies is novel, the presence of thylakoid-like membranes and a central space containing a densely stained inclusion is consistent with three possible identities that differ by the degree of integration with the host cell: (1) the bodies are ingested (photosynthetic) prey cells that are in the earliest stages of being degraded, (2) the bodies are transient photosynthetic endosymbionts that are continuously replenished by kleptoplasty, or (3) the bodies are permanently integrated photosynthetic endosymbionts (i.e. plastids). The plausibility of each of these hypotheses is addressed below.

The orange color of these bodies is reminiscent of the plastids in some microalgae, such as dinoflagellates and diatoms that occupy the same habitats as *A. quadriverberis*. However, neither dinoflagellate theca nor diatom frustules were found associated with these bodies in any TEM sections, and the ultrastructure of the bodies was very different from the known ultrastructural diversity in the plastids of diatoms and dinoflagellates. Some cyanobacteria are known to have pale orange coloration that is similar to the orange bodies within *A. quadriverberis *[42]. These orange bodies were surrounded by two tightly compressed inner membranes and sac-like vesicles. Whereas typical food bodies show degrees of being digested by cellular enzymes, nearly all of the pigmented bodies observed were completely intact in all of the cells we observed (n = 70), suggesting that they are constant fixtures of the host cell cytoplasm.

Primary endosymbiosis, involving a photosynthetic prokaryote within a eukaryotic cell, results in three surrounding membranes: two cyanobacterial inner membranes and a third, outer phagosomal membrane. Green algae/land plants, red algae, and glaucophytes possess primary plastids [43-45]. Two membranes surround the plastids of green algae and red algae, and the third outer phagosomal membrane is inferred to have been lost [43-46]. Secondary endosymbiosis occurs through the engulfment, integration and maintenance of either a green or red alga by a predatory eukaryote. This process produced the plastids of cryptomonads, haptophytes, stramenopiles, dinoflagellates, apicomplexans, and euglenids [43-45]. Two different lineages of cercozoans have independently acquired plastids through endosymbiosis: (1) chlorarachniophytes have secondary plastids derived from green algae [45,47] and (2) *Paulinella chromatophora *has primary plastids derived from cyanobacterial prey [48-50].

Like in *Paulinella *and the cyanelles of glaucophytes, the ultrastructure of the pigmented bodies within *A. quadriverberis *is most consistent with the ultrastructure of free-living cyanobacteria, suggesting an independent primary endosymbiotic origin [44,48-52]. For instance, TEM sections through the pigmented bodies demonstrated a mode of division that is similar to division described in the cyanelles of *Cyanophora paradoxa *[53] (Figure 5C). Moreover, the thylakoids in the endosymbionts of *P. chromatophora*, the cyanelles of glaucophytes, and coccoid photosynthetic cyanobacteria are unstacked and arranged concentrically around the periphery of the cell [48,54,55]. A similar arrangement was observed in the pigmented bodies of *A. quadriverberis *(Figure 5A–C), although the majority of the thylakoids projected inward towards the core of the body. The central area within the pigmented bodies of *A. quadriverberis *resembled the pyrenoids in the cyanelles of *Glaucocystis nostochinearum *[55].

The thylakoid-free core of the pigmented bodies also contained polygonal viral particles. TEM sections through these particles demonstrated complete tailed phages similar to those known to infect cyanobacteria [56-58] (Figure 5G). Viral particles similar to those described in the pigmented bodies of *A. quadriverberis *have also been described in the same region in the plastids of other eukaryotes, such as the "polyhedral bodies" in the primary endosymbionts of *P. chromatophora *[48], the cyanelles of the glaucophyte *Gloeochaete wittrockiana *[55], and the free-living photosynthetic cyanobacterium *Nostoc punctiforme *[54]. Two other important characters that have been used to infer a cyanobacterial origin for primary plastids are: (1) the presence of phycobilisomes and (2) the presence of a peptidoglycan wall [48,49,51]. However, as previously mentioned, neither phycobilisomes nor a peptidoglycan layer was present in the orange bodies in *A. quadriverberis*.

## Conclusion

Our characterization of *A. quadriverberis *n. gen. et sp. demonstrates several novel features within the Cercozoa, such as four homodynamic flagella, densely distributed linear rows of orange muciferous bodies, and putative endosymbionts with an enigmatic overall structure. The discovery of this highly distinctive lineage underscores how poorly we understand the actual cellular diversity of cercozoans and, potentially, represents one of the few independent cases of primary endosymbiosis within the Cercozoa and beyond. Although endosymbioses are known to have occurred many different times independently, the transformation of endosymbionts into organelles is considered to be much less common [59]. In order to more confidently infer the origin of the pigmented bodies in *A. quadriverberis*, experiments involving autofluorescence and the amplification of plastid molecular markers (e.g. 16S rDNA and *psb *genes) could be performed [50]. These studies will be hampered mainly by the scarcity and unpredictability of finding these cells in natural samples. Nonetheless, additional studies on *A. quadriverberis *and its putative endosymbionts will enable us to better understand the extent of endosymbiosis across the tree of eukaryotes and the convergent processes associated with the establishment and integration of endosymbionts within eukaryotic cells.

## Taxonomic descriptions

### Taxonomic treatment for *Auranticordis quadriverberis*

Phylum Cercozoa [60]

### Genus *Auranticordis *gen. nov. Chantangsi, Esson and Leander 2008

#### Diagnosis

Uninucleate tetraflagellates; four recurrent flagella inserted subapically and bundled together within a ventral longitudinal groove; all flagella about one cell length; cell shapes are prominently lobed, ovoid or heart-shaped; nucleus at anterior end of cell, with nucleoli; no cell wall or test; minute orange muciferous bodies distributed in linear arrays over the entire cell; cytoplasm with pale orange pigmented bodies, usually concentrated at the anterior end; corrugated cell surface; black inclusions usually present at anterior part of the cell; locomotion by slow gliding; cell deformations possible; marine habitat.

#### Type species

*Auranticordis quadriverberis*.

#### Etymology

Latin *aurantium*, n. orange; L. *cordis*, n. heart. The generic name reflects two characteristic features of this taxon: orange cell coloration and inverted heart-shaped cells.

### Species *Auranticordis quadriverberis *spec. nov. Chantangsi, Esson and Leander 2008

#### Description

Cell shape ovoid, prominently lobed or inverted heart-shaped; cell size 35–75 μm long, 25–70 μm wide; four homodynamic flagella, inserted subapically and bundled within a ventral longitudinal groove; anterior nucleus with nucleoli; bright orange coloration caused by linear rows of minute orange muciferous bodies; corrugated cell surface with about 80 longitudinal ridges; no cell wall or test; cytoplasm with 2–30 pale orange pigmented bodies; black inclusions usually present at anterior part of the cell; locomotion by slow gliding. Small subunit rRNA gene sequences [GenBank:EU484393 and GenBank:EU484394].

#### Type locality

Tidal sand-flat at Spanish Banks, Vancouver, British Columbia, Canada. The specimen was found during March and May, 2007.

#### Hapantotype

Both resin-embedded cells used for TEM and cells on gold sputter-coated SEM stubs have been deposited in the Beaty Biodiversity Research Centre (Marine Invertebrate Collection) at the University of British Columbia, Vancouver, Canada.

#### Iconotype

Figures 1B, 1F, 1H and 6.

#### Type locality

Spanish Banks, Vancouver, BC, Canada (39°28' N, 74°15' W).

#### Habitat

Marine sand.

#### Etymology

The etymology for the specific epithet, Latin *quattuor*, four; L. *verberis*, n. whip. The specific epithet reflects the presence of four flagella.

## Methods

### Sampling and light microscopy (LM)

Sand samples were collected from Spanish Banks, Vancouver, BC, Canada in March 2007. Organisms were extracted from the sand samples through a 48 μm mesh using a melted seawater-ice method described by Uhlig [61]. Briefly, 2–3 spoons of sand samples were placed into an extraction column wrapped with a 48 μm mesh. Two to three seawater ice cubes were then put on top of the sand samples and left to melt over several hours. The organisms of interest were separated through the mesh and concentrated in a Petri dish that was filled with seawater and placed underneath the extraction column. The Petri dish containing the organisms was then screened using a Leica DMIL inverted microscope. Cells were individually isolated and placed on a slide for light microscopy using phase contrast and differential interference contrast (DIC) microscopy with a Zeiss Axioplan 2 imaging microscope connected to a Leica DC500 color digital camera.

### Scanning electron microscopy (SEM)

Twenty-five cells of *Auranticordis quadriverberis *were individually isolated and placed into a small container covered on one side with a 10-μm polycarbonate membrane filter (Corning Separations Div., Acton, MA, USA). The samples were pre-fixed in the container with OsO_4 _vapor for 30 min at room temperature and subsequently post-fixed for 30 min with a mixture of 8% glutaraldehyde and 4% OsO_4_, giving a final concentration of 2.5% glutaraldehyde and 1% OsO_4_. The organisms were then washed three times in filtered seawater to remove the fixative and dehydrated through a graded series of ethanol. Dehydrated samples were critical point dried with CO_2 _using a Tousimis Samdri 795 CPD (Rockville, MD, USA). Dried filters containing the cells were mounted on aluminum stubs and then sputter coated with gold (5 nm thickness) using a Cressington high resolution sputter coater (Cressington Scientific Instruments Ltd, Watford, UK). The coated cells were viewed under a Hitachi S4700 scanning electron microscope.

### Transmission electron microscopy (TEM)

Two individual cells of *Auranticordis quadriverberis *were prepared separately. Each cell was pre-fixed with 2% (v/v) glutaraldehyde (in unbuffered seawater) at room temperature for 1 h. Cells were then washed three times in filtered seawater and post-fixed with 1% (v/v) OsO_4 _(in unbuffered seawater) for another 1 h at room temperature. Fixed cells were then washed three times in filtered seawater and were dehydrated through a graded series of ethanol. Infiltration was performed with acetone-resin mixtures (acetone, 2:1, 1:1, 1:2, Epon 812 resin) and individually flat embedded in Epon 812 resin. The resin containing the cell(s) was polymerized at 65°C for one day and sectioned with a diamond knife on a Leica EM-UC6 ultramicrotome. The sections were collected on copper, formvar-coated slot grids and stained with uranyl acid and lead citrate (Sato's lead method) [62,63]. TEM micrographs were taken with a Hitachi H7600 transmission electron microscope.

### DNA extraction and PCR amplification

Five cells were individually isolated and washed three times in autoclaved seawater. DNA was extracted using the protocol provided in the Total Nucleic Acid Purification kit by EPICENTRE (Madison, WI, USA). Polymerase chain reaction (PCR) was performed in a thermal cycler using puReTaq Ready-To-Go PCR beads (GE Healthcare Bio-Sciences, Inc., Québec, Canada). The forward (PF1: 5'-GCGCTACCTGGTTGATCCTGCC-3') and reverse (R4: 5'-GATCCTTCTGCAGGTTCACCTAC-3') primers for amplifying SSU rDNA were added into the tube with the final reaction volume of 25 μl. The thermal cycler was programmed as follows: hold at 94°C for 4 min; 5 cycles of denaturation at 94°C for 30 sec, annealing at 45°C for 1 min, and extension at 72°C for 105 sec; 35 cycles of denaturation at 94°C for 30 sec, annealing at 55 °C for 1 min, and extension at 72 °C for 105 sec; and hold at 72 °C for 10 min. PCR products corresponding to the expected size were separated by agarose gel electrophoresis, cleaned using the UltraClean™ 15 DNA Purification Kit (MO BIO Laboratories, Inc., CA, USA). The cleaned DNA was cloned into pCR2.1 vector using the TOPO TA Cloning^® ^kits (Invitrogen Corporation, CA, USA). Plasmids with the correct insert size were sequenced using BigDye 3.1 and the vector forward and reverse primers, and an internal primer (525F: 5'-AAGTCTGGTGCCAGCAGCC-3') with an Applied Biosystems 3730S 48-capillary sequencer.

The above processes was repeated on one additional cell of *Auranticordis quadriverberis *that were sampled and isolated at different times, in order to assure authenticity of the obtained sequences. Complete sequences of the SSU rDNA from the two different isolates were deposited into GenBank [GenBank:EU484393 and GenBank:EU484394].

### Sequence alignment and phylogenetic analyses

Sequences were assembled and edited using Sequencher™ (version 4.5, Gene Codes Corporation, Ann Arbor, Michigan, USA). Acquired sequences were initially identified by BLAST analysis. New SSU rDNA sequences derived from two different isolated of *Auranticordis quadriverberis *were aligned with ClustalW [64] using the MEGA (Molecular Evolutionary Genetics Analysis) program version 4 [65] and further refined by eye using MacClade [66]. Three multiple sequence alignments were created: (1) a 69-taxon global alignment comprising sequences of representatives from all major eukaryotic groups (1,134 unambiguous sites: data not shown); (2) a 126-taxon cercozoan alignment consisting of cercozoan representatives and extensive environmental sequences (981 unambiguous sites: data not shown); and (3) a 32-taxon cercozoan alignment excluding the shorter and unrelated environmental sequences (1,526 unambiguous sites). All gaps were excluded from the alignments prior to phylogenetic analyses. The alignment files are available upon request.

MrBayes version 3.1.2 was used to perform Bayesian analyses on all three datasets [67,68]. Two parallel runs were carried out on 2,000,000 generations with the four Markov Chain Monte Carlo (MCMC) chains – 1 cold chain and 3 heated chains – and sampling every 50^th ^generation (tree). The first 2,000 trees in each run were discarded as burn-in. Branch lengths of the trees were saved.

Maximum likelihood analyses were performed on all three datasets using PhyML [69]. Input trees for each dataset were generated by BIONJ with optimisation of topology, branch lengths, and rate parameters selected. The General Time Reversible (GTR) model of nucleotide substitution was chosen. The proportion of variable rates and gamma distribution parameter were estimated from the original dataset. Eight categories of substitution rates were selected. PhyML bootstrap trees with 100 bootstrap datasets were constructed using the same parameters as the individual ML trees.

### Sequence availability

The SSU rDNA nucleotide sequences included in 32-taxon analyses for this paper are available from the GenBank database under the following accession numbers: *Allas diplophysa *[GenBank:AF411262], *Auranticordis quadriverberis *[GenBank:EU484393 and GenBank:EU484394], *Bodomorpha minima *[GenBank:AF411276], *Bodomorpha *sp. [GenBank:DQ211596], *Cercomonas plasmodialis *[GenBank:AF411268], *Cryothecomonas aestivalis *[GenBank:AF290539], *Dimorpha*-like sp. [GenBank:AF411283], *Ebria tripartita *[GenBank:DQ303922], *Euglypha rotunda *[GenBank:AJ418784], *Exuviaella pusilla *[GenBank:DQ388459], *Gymnophrys cometa *[GenBank:AF411284], *Heteromita globosa *[GenBank:U42447], *Lecythium *sp. [GenBank:AJ514867], *Massisteria marina *[GenBank:AF174372], *Metopion*-like sp. [GenBank:AF411278], *Paulinella chromatophora *[GenBank:X81811], *Proleptomonas faecicola *[GenBank:AF411275], *Protaspis grandis *[GenBank:DQ303924], *Pseudodifflugia *cf. *gracilis *[GenBank:AJ418794], *Pseudopirsonia mucosa *[GenBank:AJ561116], *Rigidomastix*-like sp. [GenBank:AF411279], *Spongomonas minima *[GenBank:AF411280], *Thaumatomastix *sp. [GenBank:AF411261], thaumatomonadida environmental sample [GenBank:EF023494], *Thaumatomonas coloniensis *[GenBank:DQ211591], *Thaumatomonas seravini *[GenBank:AF411259], uncultured eukaryote [GenBank:AB252750], uncultured eukaryote [GenBank:AB252755], uncultured eukaryote [GenBank:AB252756], uncultured eukaryote [GenBank:AB275058], and uncultured marine eukaryote [GenBank:DQ369017].

## Authors' contributions

CC and BSL conceived and designed the experiments. CC, HJE, and BSL performed microscopical studies. CC conducted the molecular studies, the sequence alignments, and phylogenetic analyses. CC and BSL analyzed the data, drafted the manuscript, and wrote the paper. All authors have read and approved the final manuscript.
